# Central Dental Association of Northern New Jersey

**Published:** 1888-02

**Authors:** 


### CENTRAL DENTAL ASSOCIATION OF NORTHERN NEW JERSEY.

A regular monthly meeting was held at Newark, Monday evening, December 19th, President Watkins in the chair.

Dr. G. W. Weld, of New York, read a paper on the extraction of the sixth year molars, illustrated by drawings and models. (See page 75.)

*Dr. W. D. Tenison*—Mr. President, Dr. Weld's paper is, so far as it goes, very scientific, and probably logical, but I must take exceptions to some of its statements regarding extraction of the sixth year molars. I do not believe it wise to attempt to formulate any universal rule. In a practice of over twenty-five years I have had opportunities for seeing a great deal of the results of extraction, and while many of them are deplorable, in other cases much of benefit has been derived. When I advise the extraction of any of the sixth year molars I advise the extraction of all four of them, no matter whether they are decayed or not, provided both jaws are normal.

The older practitioners know very well that there have been monuments of skill built up in the mouths of their patients in the restoration of sixth year molars, after extensive decay of those teeth, and that in after years they have broken away and been lost. In cases like the one of which a model has been presented, with a perfect articulation of the teeth, I think it would be a crime to extract, but, unfortunately, we do not always find that condition. Where the sixth year molars indicate a tendency to extensive decay and the remaining teeth are in a reasonably perfect condition, I believe it would be proper to extract them. I do not approve of extraction at eight or nine years of age. They should be removed when the second molars are beginning to make their appearance. I will say further, that the gentlemen who object to extraction of the sixth year molars usually produce models of the worst cases. That is not fair. If you look at this mouth I think you will admit that it would be difficult to find one more perfect. (Model shown.) The young man whose mouth this represents is now eighteen years of age. The sixth year molars were extracted at the age of twelve. Unfortunately I have not models of the mouth as it appeared before the extraction of the teeth.

Here are two models of the upper jaw of a little girl for whom I extracted the sixth year molars. The lower models were lost a year ago. These two superior models show the condition immediately after the extraction of the teeth, and nine months subsequently. There is no tipping of the teeth in these cases.. I do not pretend to say that that never occurs; but the question arises, whether the condition we would find in after years, if those teeth were left in the mouth, would not be a great deal worse than that which follows their extraction? You cannot safely follow any iron-clad rule in this matter. I have among my patients families of children now grown up, in whose mouths the results of the extraction and non-extraction of these teeth are shown in strong contrast. When the elder children came into my hands I had not the experience in this matter that I acquired in after years, and I neglected to extract the sixth year molars, as I did in the cases of the younger children; and in the cases where the sixth year molars were extracted there are now good sets of teeth, while the others are constantly under my care. I have always regretted that I did not extract in the earlier cases as well as in the later.

Dr. Weld has ingeniously introduced the diagram of a mechanical arch to prove that it is a mistake to extract teeth, but we do not find such an arch as that in the mouth, and the pressure is not the same when we bite.

*Dr. Weld*—The principle is the same.

*Dr. Tenison*—That is a principle of mechanics in connection with building a house, but it hardly applies to dentistry.

*Dr. Osmun*—Mr. President, I am glad that Dr. Weld has given us this paper, but after all is said and written on both sides of this question, the real kernel of wheat that is sifted out is that this matter must be left to educated judgment and experience. I have seen cases so plain that there was no second thought about the propriety of extracting the sixth year molars; it did not require any consideration to determine that as the proper thing to do; and I have seen other cases just the reverse. Taking the children of the day, generally, as they come to us in this nervous generation, you will find that the sixth year molars are usually in a decayed, decalcified condition. My experience has been that when those teeth are filled, devitalization usually occurs very soon after, and when you resort to devitalization in order to insert filling, the teeth may remain from three to five years, and then they begin to go the way of all flesh. When children come to us after the age of nine or ten years, with the twelfth year molars in place, thoroughly erupted and solidified, if we remove the sixth year molars tipping of the other teeth will result. But if extraction be done prior to the eruption of the second molars, or just as they are coming through the gum, in nine cases out of ten excellent results will follow. I have a number of such cases where the articulation is as perfect as it would be with thirty-two teeth in the mouth.

Another point should be borne in mind in this connection, which is the fact that the third molar is scarcely ever a very solid or well organized tooth, but is usually soft, decalcified and very susceptible to decay. In many cases they are decayed almost as soon as they put in an appearance in the mouth. Now, if the operator has been premature in extracting the first molars, and the third molars are in a bad condition, the second molars being good, he has only one molar tooth left to rely on for mastication, for in such cases I have found it almost impossible to save the third molars.

*Dr. Baldwin*—I admit that the sixth year molar is a very weak tooth, and many dentists think that because it is weak it should be removed. I am very much inclined to fall back on the old theory that if it were not best that it should be there it would not have been put there, and there must be some good reason for its removal. Parents do not know how to distinguish between the sixth year molar and a deciduous tooth, and many physicians are equally ignorant. Oftentimes they tell a patient to have a sixth year molar extracted, when, if they really knew it was a permanent tooth, and one that would be useful, they would hesitate, and the tooth might be saved. I think that we can preserve and retain the sixth year molars in a large majority of cases, so many that the exceptions but prove the rule.

*Dr. Weld*—I do not wish to be understood as holding that there are no exceptions to the principle involved in the illustrations I have presented this evening. My friend, Dr. Tenison, has brought an exceptional case here to support his side of the argument, one in which there has been a complete translation, and no rotation, and it is one case in ten thousand. It is phenomenal that in six years, perhaps less, there was a complete translation with hardly any rotation whatever. The cases which have come under my own observation, and that of others who are older than I am in practice, are those in which the second molars have tipped forward, and there is rotation, accompanied with complete or incomplete translation, resulting in disarticulation and loss of masticating power.

*Dr. Tenison*—Probably the doctor would admit, if I can show several cases of that kind in the next six months or so, that that is not one case in thousands. I am sorry that Dr. Weld has not seen a case of this kind before. I have seen a number of them. But I do not bring this forward to prove that the sixth year molars should always be extracted. I do not advocate that, but that our judgment should be used as the cases come up.

I do not condemn the retention of the sixth year molars; I simply say that those who advocate it produce the cases of extraction that show the worst results. They may say I show the best. I believe we should show both sides to the younger members of the profession, and teach them to use their judgment in each individual case, as to whether the sixth year molars should be extracted.

I have extracted the second bicuspids. My daughter had a most magnificently articulated set of teeth. I had refrained from extracting, as I thought it bad practice in such cases, and at the age of sixteen every one of her teeth showed traces of decay in the approximal surfaces. I did not file them apart, but extracted the second bicuspids, and by the aid of plates opened the teeth. Naturally, her teeth were very frail. Now, at the age of twenty-one, she has a very fair set of teeth. I believe she would have lost most of them if I had not done as I did. I would have given thousands of dollars rather than have been obliged to extract those teeth. I never, in my life, saw a better articulated set than they were at that time. But it was a question of saving or losing all her teeth, and the extraction of the bicuspids at that age did not leave as much space as the extraction of the sixth year molars would have done.

*Dr. Palmer*—Mr. President, there is only one point to which I desire to refer in connection with the paper that has been read. Judging from Dr. Tenison's last remarks, it is not in his opinion entirely a question of the exercise of one's judgment regarding the extraction of the first permanent molars, but as to whether some teeth should be removed that may relieve the crowded condition; and he extracts a molar or a bicuspid, whichever will best serve the purpose. Another point has been, I think, lost sight of in the discussion, and it is, that if any of the first permanent molars are extracted, all should be taken.

*Dr. Evans*—I do not think it is always judicious to extract the four sixth year molars. Very often we have a large arch in the lower jaw, while in the upper jaw the teeth are much crowded. In such a case it is not necessary to extract from the lower jaw, where the second molars are present. We relieve the pressure of the teeth by extracting in one jaw, and in a measure overcome the difficulty.

*Dr. Tenison*—I did not intend to make the extraction of the four first molars a universal rule. I think it is a matter of judgment, and I agree with him that in some cases it is proper to extract only in the upper or the lower jaw, according to the condition found. The development of one jaw may be more perfect than the other. When I advised the removal of the four molars if any were extracted, I was taking it for granted that the two jaws were developed about equally.

Upon motion, the subject was passed.

